# Fellow-Workers and Their Doings

**Published:** 1914-03-14

**Authors:** 


### ROUND THE WORLD.

### Fellow-Workers and their Doings.

[The Editor Invites Early Information and Contributions Marked "Fellow-Workers."]

Mr. Ernest Hey Groves, M.S. Lond., F.R.C.S., whose long-continued researches on the operative treatment of fractures has gained him the honour of a Hunterian Lectureship, is surgeon to the Bristol General Hospital and an old "Bart.'s" man. Although his contributions to medical literature have been many, he is perhaps best known where students are gathered together as the author of his invaluable "Synopsis of Surgery."

Mr. J. L. Birley, who is an Oxford medical graduate as well as an M.R.C.S., L.R.C.P., has succeeded to the responsible and valuable experience of the resident assistant physiciancy at St. Thomas's Hospital. This post stands alone, as does that of the theatre resident assistant surgeoncy at the same hospital, no quite similar post being found at any other Metropolitan institution. Both may be looked upon as likely stepping-stones to important appointments on the general staff.

Mr. William Lang, F.R.C.S., who has lately retired from the staff of the Middlesex Hospital, was appointed ophthalmic surgeon there in 1881, and on his retirement was the "Father of the Staff." This popular ophthalmologist did pioneer work in this country in showing and teaching that many inflammatory eye conditions are secondary to sepsis in the gums and elsewhere. It is interesting to recall that the appointment of a dental surgeon at the Royal London Ophthalmic Hospital (Moorfields) was largely due to his advice.

Dr. Killick Millard, M.D., D.Sc., who has just delivered the Chadwick lectures on vaccination, is the medical officer of health for Leicester, which is the Mecca of anti-vaccinationists. In his lectures, which therefore had a special interest, Dr. Millard was dogmatic as to the absolute benefits of vaccination as a prevention of a loathsome disease, at the same time emphasising the efficiency of sanitation in reducing smallpox mortality.

Dr. Thomas Lewis, M.D., F.R.C.P., D.Sc., the director of the cardiological department at University College Hospital, is engaged upon a very interesting investigation, the object of which is to demonstrate as completely as possible the vascular supply of the heart itself. It is felt that an understanding of the blood-supply of the heart is an essential preliminary to further investigation of the nodal causes of cardiac arrhythmia. To this end Dr. Lewis inserts cannulæ into each orifice of the excised heart, which is then transfused with saline solution to remove all blood. After this, formalin is injected, a proceeding which results in the curling up of the numerous small valves in the veins. The way is then clear for the injection of a red and blue plaster-of-Paris preparation, which clearly shows up even the most minute vessels.

At St. Thomas's Hospital Mr. Edred Corner, F.R.C.S., is especially investigating surgery of injured and diseased joints. He is obtaining very good results from his operative treatment of old ankylosed cases of Pott's fracture. One of the chief points of Mr. Corner's operation for this ailment is the interposition of fascia between the bony surfaces. Mr. Corner is the author of several well-known text-books.

Dr. W. Scarisbrick, the new tuberculosis officer to the Southend-on-Sea Borough Council, was formerly a Liverpool University student, and holds the degrees of M.D., B.Sc., M.R.C.S., and L.R.C.P. Lond., and D.P.H. Liverpool. He has lately been medical superintendent to the National Sanatorium at Benenden, Kent, and was previously assistant medical officer of health and school medical officer at Southend.

Mr. E. G. Schlesinger, F.R.C.S., who has had a brilliant career at Guy's Hospital Medical School, where, amongst other awards, he gained a science scholarship and the Michael Harris prize in anatomy, has been elected to one of the British Medical Association Research Scholarships.

Dr. Arthur Walmsley Stott, M.R.C.P., has just been appointed first assistant to Dr. Thomas Lewis in the electro-cardiological department at University College Hospital. Dr. Stott is an old Rugby and "Bart.'s" man, and is cardiologist to the Royal Hospital for Diseases of the Chest. He is also a demonstrator of pathology and chief assistant to the children's department at St. Bartholomew's Hospital, where he was formally a student.

Mr. Eric Pearce Gould, M.Ch., F.R.C.S., has lately concluded an interesting research at the Middlesex Hospital in connection with the pathology of mesenteric cysts. One of his conclusions is that several of the cases of this condition recorded should really have been diagnosed as chronic tubercular abscess, an observation that is supported by the fact that several supposed cysts of the mesentery, which came under his notice on careful investigation proved to be of tubercular origin. Mr. Pearce Gould, who is a son of the well-known Middlesex Hospital surgeon, has now gone to Berlin, with the special object of working in the pathological institutions there.

The new assistant medical superintendent of the Paddington Infirmary, Mr. H. Wilson White, is a graduate of the University of Ireland, and has had much experience of institutional work, having served on the staff of the Manor and Long Grove Asylums at Epsom, and latterly at the Westminster Union Infirmary. Mr. White was also for some time resident at the Metropolitan Hospital.

Dr. J. Bright Banister, who has just been promoted from registrar to out-patients' surgeon at the Chelsea Hospital for Women, is a member of the staff of the Queen Charlotte's Hospital, and holds the double appointment of registrar and tutor in the obstetric department at Charing Cross Hospital, where he pursued his medical studies after leaving Cambridge. Mr. Banister is an M.D. Cantab., M.R.C.P. Lond., and an F.R.C.S. Edin.

Mr. C. A. Joll, F.R.C.S., who has been appointed assistant surgeon to the Royal Free Hospital (School of Medicine for Women), qualified M.B., B.S. Lond. in 1909, and not 1911, as was inadvertently stated.

